# A randomised factorial trial of sequential doxorubicin and CMF *vs* CMF and chemotherapy alone *vs* chemotherapy followed by goserelin plus tamoxifen as adjuvant treatment of node-positive breast cancer

**DOI:** 10.1038/sj.bjc.6602355

**Published:** 2005-01-25

**Authors:** S De Placido, M De Laurentiis, M De Lena, V Lorusso, A Paradiso, M D'Aprile, G Pistillucci, A Farris, M G Sarobba, S Palazzo, L Manzione, V Adamo, S Palmeri, F Ferraù, R Lauria, C Pagliarulo, G Petrella, G Limite, R Costanzo, A R Bianco

**Affiliations:** 1 Department Molecular and Clinical Oncology and Endocrinology, University Federico II, Via S Pansini 5, Napoli 80131, Italy; 2 See Appendix A for a complete list of co-authors and Institutions

**Keywords:** adjuvant therapy, anthracyclines, breast cancer, chemoendocrine treatment, premenopausal

## Abstract

The sequential doxorubicin → CMF (CMF=cyclophosphamide, methotrexate, fluorouracil) regimen has never been compared to CMF in a randomised trial. The role of adding goserelin and tamoxifen after chemotherapy is unclear. In all, 466 premenopausal node-positive patients were randomised to: (a) CMF × 6 cycles (CMF); (b) doxorubicin × 4 cycles followed by CMF × 6 cycles (A → CMF); (c) CMF × 6 cycles followed by goserelin plus tamoxifen × 2 years (CMF → GT); and (d) doxorubicin × 4 cycles followed by CMF × 6 cycles followed by goserelin plus tamoxifen × 2 years (A → CMF → GT). The study used a 2 × 2 factorial experimental design to assess: (1) the effect of the chemotherapy regimens (CMF *vs* A → CMF or arms a+c *vs* b+d) and (2) the effect of adding GT after chemotherapy (arms a+b *vs* c+d). At a median follow-up of 72 months, A → CMF as compared to CMF significantly improved disease-free survival (DFS) with a multivariate hazard ratio (HR)=0.740 (95% confidence interval (CI): 0.556–0.986; *P*=0.040) and produced a nonsignificant improvement of overall survival (OS) (HR=0.764; 95% CI: 0.489–1.193). The addition of GT after chemotherapy significantly improved DFS (HR=0.74; 95% CI: 0.555–0.987; *P*=0.040), with a nonsignificant improvement of OS (HR=0.84; 95% CI: 0.54–1.32). A → CMF is superior to CMF. Adding GT after chemotherapy is beneficial for premenopausal node-positive patients.

Adjuvant therapy of breast cancer is one of the most successful treatment strategies in oncology. Hundreds of randomised clinical trials of adjuvant therapy conducted worldwide have demonstrated the efficacy of many therapeutic options, both hormonal and cytotoxic. Not all data coincide, which makes it difficult to select the best adjuvant combination for each patient. In an attempt to elucidate controversial issues, the Early Breast Cancer Trialists' Collaborative Group (EBCTCG) (1996, 1998a, 1998b) periodically reviews the randomised evidence. However, the last update of the meta-analysis leaves several important questions unanswered (2000a, 2000b).

First, anthracycline-based polychemotherapy is, on average, more effective than CMF-like (CMF=cyclophosphamide, methotrexate, fluorouracil) regimens, producing a further 15% reduction in the annual odds of death. However, uncertainty remains as to what is the optimal anthracycline-based regimen. Different anthracycline-based regimens have become standard in Europe, the USA and Canada. However, for some of these regimens, there is no clear proof of superiority over the CMF. For instance, a sequential regimen consisting of four courses of doxorubicin followed by various courses of CMF has gained widespread acceptance as a standard regimen in Europe. This was fuelled by the results of a randomised trial by Bonadonna *et al* (Buzzoni *et al*, 1991; Bonadonna *et al*, 1995), in which this sequential regimen compared favourably with a regimen alternating doxorubicin and CMF courses. However, the superiority of the Bonadonna regimen over the classical CMF regimen has never been demonstrated.

Second, the optimal use of hormonal therapy in premenopausal women remains one of the most controversial issues. For this group of patients, ovarian ablation and tamoxifen are both effective adjuvant therapies (EBCTCG 1996, 1998a, 1998b, 2000a, 2000b). However, it is unclear as to whether ovarian suppression is beneficial after chemotherapy, and whether the combination of tamoxifen plus ovarian suppression is more effective than either treatment alone, particularly in patients who also receive chemotherapy.

In 1991, the *Gruppo Oncologico Centro-Sud-Isole* (GOCSI), an Italian Cooperative Group, started a randomised clinical trial of adjuvant therapy for premenopausal early breast cancer patients. The trial, labelled MAM1, was designed with a factorial scheme (1) to compare the efficacy of a sequential doxorubicin → CMF regimen *vs* CMF and (2) to evaluate the benefit of adding tamoxifen plus ovarian suppression obtained with goserelin after chemotherapy. Here, we report the results of the MAM1 trial after a median follow-up of 6 years.

## PATIENTS AND METHODS

This trial was open for patient accrual between September 1991 and December 1996. Its objective was to treat premenopausal women affected by primary adenocarcinoma of the breast with axillary lymph nodes involvement and no distant metastases (T0–T3, N1/2, M0). Premenopausal status was indicated by the regular occurrence of menses at the time of the randomisation. A premenopausal hormonal profile was required for patients with up to 6 months of amenorrhoea and for patients who had previously undergone hysterectomy for benign disease.

Primary therapy consisted of removal of the entire cancer by a segmental mastectomy (quadrantectomy) plus axillary dissection or a modified radical mastectomy with no gross or microscopic invasive tumour at the resection margin. Required laboratory data were limited to an initial bilirubin level within 1.5 times the institutional upper normal limit (UNL): AST ⩽2.0 × UNL, creatinine level ⩽1.5 × UNL and, before each cycle of chemotherapy (including the first), leucocytes count ⩾4000 ml^−1^ and platelet count ⩾100 000 ml^−1^. Eligible patients also had pretreatment chest radiographs, bone scan, liver ultrasounds and ECGs. Steroid receptor analysis was carried out by immunohistochemistry with a cutoff of 10% of cells with specific staining. All patients provided written informed consent meeting all national and institutional guidelines.

Treatment was delivered on an outpatient basis, starting within 6 weeks from primary surgery.

Randomisation was performed centrally at the GOCSI Data Operations unit located in Naples, Italy, upon fax request and verification of selection criteria by the central data manager. Allocation was performed by computerised minimisation procedure. After balancing for centre and lymph node involvement (1–3 *vs* ⩾4 nodal metastases), patients were allocated to one of four treatment arms:

- CMF × 6 cycles (CMF).
- Doxorubicin × 4 cycles followed by CMF × 6 cycles (A → CMF).
- CMF × 6 cycles followed by goserelin plus tamoxifen × 2 years (CMF → GT).
- Doxorubicin × 4 cycles followed by CMF × 6 cycles followed by goserelin plus tamoxifen × 2 years (A → CMF → GT).

The study used a 2 × 2 factorial experimental design to assess the following two ‘factors’: (1) the effect of the chemotherapy regimens (CMF *vs* A → CMF or arms a+c *vs* b+d) and (2) the effect of adding GT upon completion of chemotherapy (arms a+b *vs* c+d).

Irrespective of study arm: doxorubicin was given at 75 mg m^−2^ every 3 weeks; CMF consisted of cyclophosphamide 100 mg m^−2^ × o.s. on days 1–14, methotrexate 40 mg m^−2^ and fluorouracil 600 mg m^−2^ both given intravenously (i.v.) on days 1 and 8 every 4 weeks; goserelin was given by subcutaneous implant of 3.6 mg every 4 weeks for 2 years (26 administrations); and tamoxifen was given per o.s. at 20 mg q.d. for 2 years.

Complete blood cell counts were obtained before each chemotherapy treatment. If the WBC count was less than 4000 ml^−1^ or the platelet count less than 100 000 ml^−1^ on day 1, chemotherapy was delayed. If after a 1-week delay these minimal levels were not achieved, dose was reduced by 25% decrements according to a prespecified scheme based on the degree of toxicity. If haematologic toxicity was present on day 8 of CMF, the dose was reduced without treatment delay.

Radiation therapy, when used, was given after completion of chemotherapy. Although recommendations regarding this technique were included in the written protocol, investigators were permitted to follow institutional guidelines.

All patients were evaluated every 3 months during years 1–3, twice annually for the next 3 years and annually thereafter. A chest X-ray and a liver ultrasound were obtained at entry, every 6 months during years 1–6 and annually thereafter. A bone scan and a mammogram were required before treatment was started, then annually during years 1–6 and every 2 years thereafter.

Disease-free survival (DFS), which was the primary study end point, was measured from study entry until local recurrence, distant relapse, contralateral breast cancer or death without relapse, whichever occurred first. Surviving patients who were disease free were censored at the date on which they were last known to be free from their primary breast cancer. The secondary end point of overall survival (OS) was measured from study entry until death from any cause; surviving patients were censored at the date of last contact.

Target accrual was 940 patients over 36 months, with the data being analysed 3 years after completion of accrual. This provided 80% power to detect an 8% absolute difference in event rate for either main effect, assuming an event rate equal to 70% in the control groups. The trial was not dimensioned to look for interactions between the two main factors. Kaplan–Meier curves with log-rank tests were used to compare the distribution of time with events. Cox's proportional hazards regressions with Wald's *χ*^2^ tests were used to model and assess the relation between DFS and OS with treatment factors, adjusting for clinical variables. The interaction between factors was assessed by the likelihood ratio test. Disease-free survival and OS analyses are on an intention-to-treat basis. All *P*-values are two-sided.

Toxicity grading used the NCI common toxicity criteria. Patient information was collected on standard study forms by the GOCSI Data Operations unit located in Naples, Italy, and entered into the GOCSI database. Data were current as of May 2002.

## RESULTS

**Figure 1:**
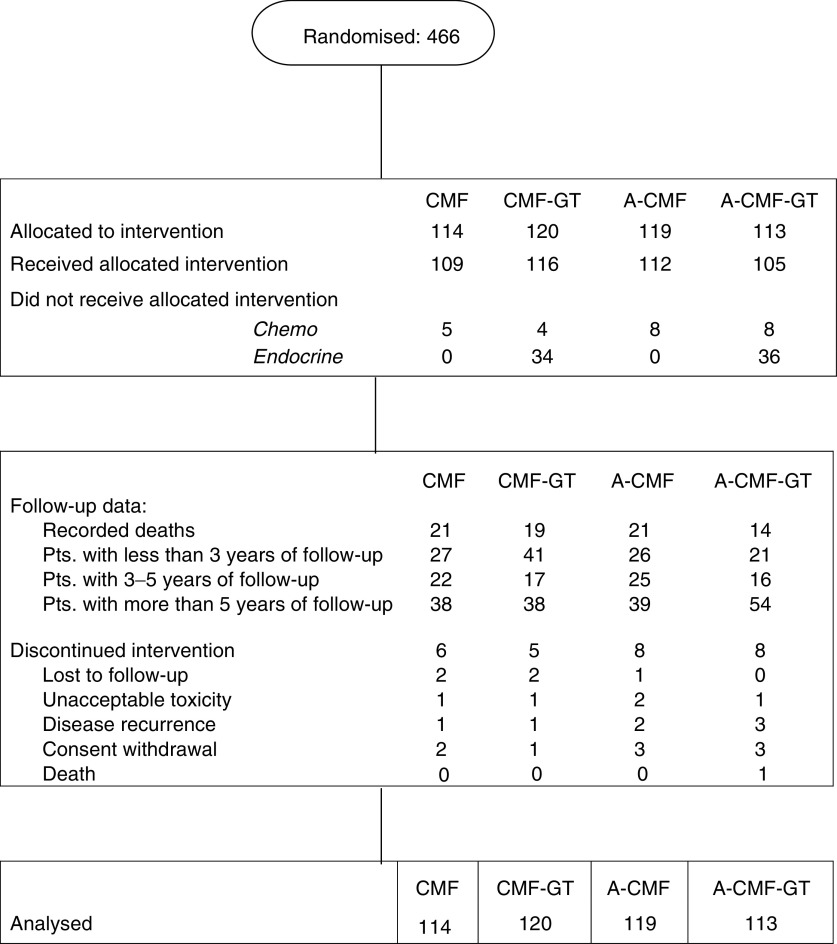
Complete patient flow according to the Consort requirements.

Between September 1991 and December 1996, 466 volunteer female patients were accrued by 19 Italian centres. This total was about half of that planned (940); however, the trial was closed for a slower-than-expected accrual rate. The complete patient flow according to the Consort requirements is reported in Figure 1. The overall compliance to the treatment was 95% for chemotherapy and 70% for endocrine therapy (Figure 1). The main characteristics of this patient population are reported in Table 1 . The median patient age was 44 years, 79% had oestrogen receptor (ER)-positive or -unknown tumours, the median number of involved lymph nodes was 3 and 44% had four or more affected axillary lymph nodes. The regimens were balanced with regard to these and all other major pretreatment variables. After a median follow-up of 72 months, there were 194 relapses and 82 deaths recorded.

### First comparison: A → CMF *vs* CMF

**Figure 2:**
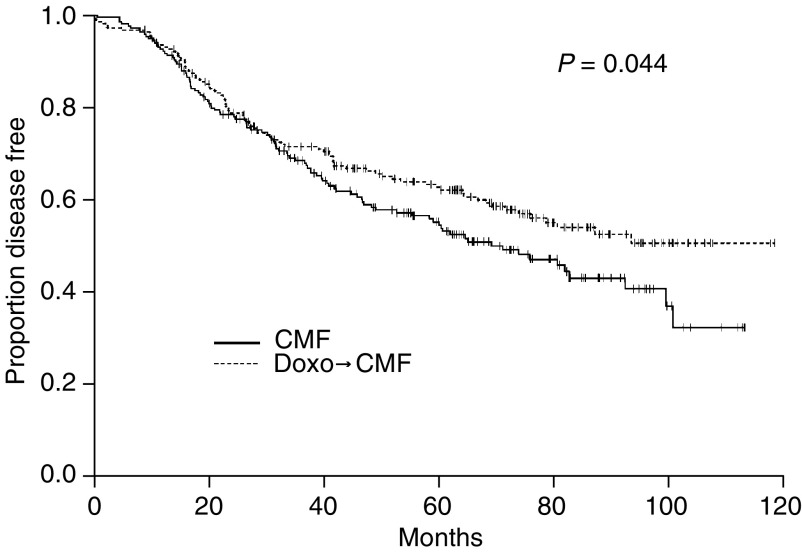
Disease-free survival curve by type of chemotherapy.

At univariate analysis, DFS was significantly prolonged for the sequential anthracycline-based regimen (arms b+d) compared with the CMF regimen (arms a+c) (Figure 2). The estimated DFS at 5 years were 65% for the sequential treatment and 54% for CMF (*P*=0.044). This effect remained statistically significant at multivariate analysis even after adjusting for the number of positive nodes (1–3 *vs* ⩾4), tumour size (⩽2 *vs* >2 cm), age (⩽35 *vs* >35) and tumour ER status and stratifying based on the GT treatment with a hazard ratio (HR)=0.740 (95% confidence interval (CI): 0.556–0.986; *P*=0.040).

The estimated OS at 5 years were 83% for A → CMF *vs* 79% for CMF. The difference was not significant at univariate log-rank test (*P*=0.26) or at Cox's multivariate analysis (HR=0.764; 95% CI: 0.489–1.193).

### Second comparison: chemo → GT *vs* chemo

**Figure 3:**
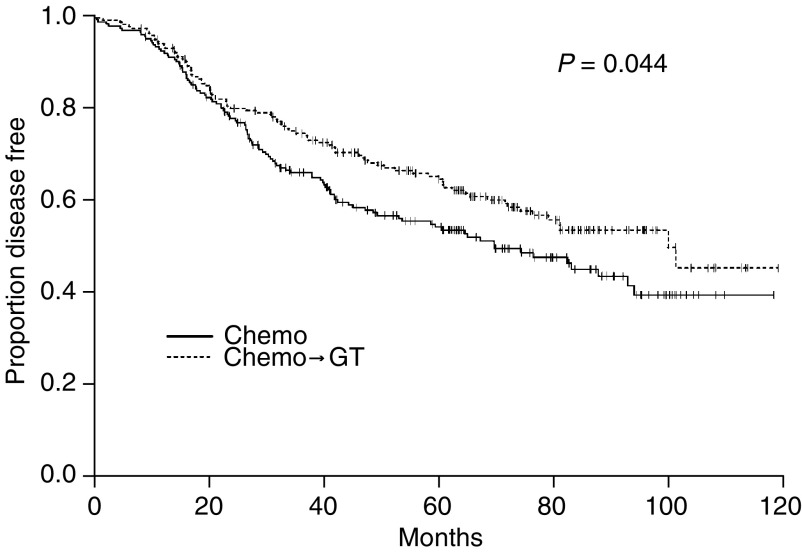
Disease-free survival curve in the presence of GT.

At univariate analysis, DFS was significantly improved by the addition of GT after chemotherapy (arms c+d) compared with chemotherapy alone (arms a+b) (Figure 3). The estimated probability of being disease free at 5 years was 64% for chemo → GT and 53% for chemotherapy alone (*P*=0.044) (Figure 3). The benefits of adding GT remained statistically significant at multivariate analysis even after adjusting for the number of positive nodes, tumour size, age and tumour ER status and stratifying by type of chemotherapy (A → CMF *vs* CMF) with a HR=0.740 (95% CI: 0.555–0.987; *P*=0.040). As expected, there was a trend toward a greater efficacy of the GT treatment in the ER-positive/ER-unknown subgroup of patients as compared with the ER-negative patients (HR: 0.73 *vs* 0.89, respectively). However, probably due to the low statistical power of the test, the interaction between GT effect and ER status was not statistically significant.

The estimated OS at 5 years were 82% for chemo → GT *vs* 80% for chemotherapy alone. The difference did not reach statistical significance at univariate log-rank test (*P*=0.48) or at Cox's multivariate analysis (HR=0.84; 95% CI: 0.54–1.32).

### Interaction between factors

This study was not designed for formal comparisons among single arms, nor for analysis of the interaction between factors. Nevertheless, for description purposes this analysis is reported. Kaplan–Meier estimates of DFS at 5 years were 0.51 for CMF, 0.56 for CMF → GT, 0.53 for A → CMF and 0.71 for A → CMF → GT. At multivariate analysis, HR was 0.86 for CMF → GT, 0.86 for A → CMF and 0.53 for A → CMF → GT as compared to the reference CMF arm. Although a lower HR was evident for the arm in which both factors (sequential anthracycline and GT) were associated, there was no statistical significant interaction between the two factors (likelihood ratio test *P*=0.55).

### Toxicity

The incidence of standard toxicity data for grades 3 to 4 during chemotherapy administration is reported in Table 2 on a per patient basis. There were no treatment-related deaths during chemotherapy. There was only one death within the first 6 months of protocol treatment; the cause of death, cerebral infarction, was considered unrelated to treatment. As expected, a higher incidence of grade 3–4 alopecia was seen for the A → CMF regimen (30 *vs* 1.7%; *P*<0.0001). Grade 3 or greater emesis was significantly more common for the anthracycline-containing regimen (8.2 *vs* 3.8%; *P*=0.048). Incidence of other adverse events was well balanced across arms.

The number of cycle delays was relatively small, thus accounting for a delivered dose intensity of more than 90% of the planned one, in each arm. Dose reductions were also infrequent with a total delivered dose of more than 95% of the planned one, in each arm.

## DISCUSSION

Our current understanding of the adjuvant treatment of breast cancer is based on more than 25 years of randomised trials. Interpretation of such an amount of data is not always straightforward and represents a challenging task for the scientific community. A formidable help in addressing controversial issues comes from the periodical meta-analysis of all randomised trials worldwide by the EBCTCG. Nonetheless, after its last update, a good deal of uncertainty remains as to what is the optimal anthracycline-based regimen and which is the best adjuvant strategy in premenopausal women with endocrine-responsive tumours. The MAM1 trial reported here attempts to answer two main questions that may contribute to elucidate both areas of controversy. As a general limitation, the trial is penalised by a half-than-expected accrual that have greatly reduced its statistical power. It may be argued that this decreases the value of the trial results. However, underpowered trials have by definition a high chance of not detecting a statistically significant difference between treatments (i.e. high chance of being false-negative trials). On the contrary, once a statistically significant difference is detected, as in the case of the MAM1 trial, the small sample size does not increase the risk that such a difference be a spurious result (i.e. a false-positive), although it affects the precision of the point estimate for the HR.

The EBCTCG overview results, published in 1998 (1998a) and updated in September 2000 (2000a), have definitely shown that anthracycline-based CT yields superior results in terms of recurrence and mortality rates when compared with CMF. However, diverse polychemotherapy regimens containing an anthracycline are currently used in clinical practice and there is no universally accepted standard regimen (Wood *et al*, 1994; Budman *et al*, 1998; Mouridsen, 1999, 2000; Piccart *et al*, 2001; Cardoso *et al*, 2002; Fumoleau *et al*, 2003). In the absence of direct comparisons between currently used anthracycline-based regimens, the definition of standard regimen should be based on a demonstrated superiority over the CMF.

In 1991, the Milan group (Buzzoni *et al*, 1991) published the first report of a randomised trial evaluating a sequential regimen based on doxorubicin followed by CMF, which was compared with a regimen alternating two cycles of CMF and one cycle of doxorubicin. The sequential therapy arm was superior, and at 5 years, the DFS rate was 61% for 179 patients with an average of nine involved lymph nodes. The results have been confirmed at 10-year follow-up (Bonadonna *et al*, 1995). This trial was designed to compare two different schedules of administration of the same drugs, based on theoretical considerations and mathematical modelling, and lacked of a standard control arm (i.e. a simple CMF arm). Nonetheless, the reported 5-year DFS for the sequential arm was judged by many in Europe as an indirect proof of the superiority of this regimen over the classical CMF, thus causing its rapid acceptance as standard treatment for node-positive patients. It was also argued that this sequential doxorubicin–CMF regimen should be the preferred way of incorporating the anthracycline in an adjuvant regimen. Indeed, according to the Norton–Simon model and based on the apparently excellent DFS in the Bonadonna trial, many oncologists have deemed the sequential regimen superior to those regimen in which the anthracycline was replacing a CMF drug (i.e. FAC/CAF (cyclophosphamide, doxorubicin, 5-fluorouracil) regimens). At that time, the MAM1 trial was promptly designed to evaluate directly, with one of its two factorial comparisons, the superiority of this sequential regimen over the classical CMF and it represents to date the only full report addressing this issue. Owing to its inherent design, with an imbalanced number of chemotherapy cycles between arms (six for CMF and 10 for ACMF), our trial cannot clarify whether the observed DFS improvement is related to the introduction of the anthracycline or to the longer chemotherapy administration in the sequential arms. Nonetheless, our results demonstrate the superiority of the sequential regimen, therefore providing experimental support to the conviction that such a regimen should be deemed as a standard anthracycline-based regimen. Also, it confirms a fair toxicity profile for this regimen, with a very manageable incidence of grade 3–4 adverse events. Given this very low toxicity profile, it may be questioned that some under-reporting may have occurred. However, the high average relative dose intensity and the high delivered total dose confirm that the regimen was well tolerated. Furthermore, both toxicity rates and delivered dose and dose intensity are consistent with what is already reported for the same regimen (Buzzoni *et al*, 1991).

The finding of our trial is corroborated by the pooled analysis of two large randomised studies comparing a similar sequential regimen, with epirubicin in substitution of doxorubicin, against CMF, very recently reported in abstract form (Poole *et al*, 2003). Consistently with the results of our trial, the combined analysis demonstrated a benefit for the sequential regimen in both relapse-free survival (HR=0.70; *P*=0.0003) and OS (HR=0.64; *P*=0.0001). With regard to the hypothesised superiority of the sequential regimen as compared to the FAC/CAF regimens, neither our trial nor the above-cited pooled analysis is designed to give information about this topic. However, a recent trial directly addressing the issue of the scheduling (sequential *vs* concurrent) failed to show any difference between these two treatment schedules (Citron *et al*, 2003), and thus the present evidence does not support the preferential use of sequential schedules.

Not all the other adjuvant anthracycline-based regimen used worldwide have equally demonstrated their superiority *vs* the CMF. In the last published EBCTCG meta-analysis (1998a), the advantage of anthracycline-based CT was found almost exclusively when a three-drug regimen was used (either CEF (cyclophosphamide, epidoxorubicin, fluorouracil) or CAF. Since the 1998 Oxford publication, four more large trials have been reported. Two of these trials tested a three-drug regimen and two a two-drug regimen. In the Intergroup Study 0102, six cycles of CAF were superior to six cycles of CMF in 2691 high-risk node-negative breast cancer patients (Hutchins *et al*, 1998). In the Danish–Swedish Breast Cancer Cooperative Group randomised 1195 breast cancer patients, nine cycles of CEF resulted in better 6-year survival in some patient subsets as compared to nine cycles of CMF, given only on day 1 of a three-weekly cycle (Mouridsen *et al*, 1999). However, in this trial, the ‘monodose’ CMF could be considered a suboptimal control. In a Belgian study (Piccart *et al*, 2001), no difference emerged between CMF and full dose EC with 50 months of median follow-up. Similarly, in the NSABP B-23 trial, no differences were found between four cycles of a two-drug regimen (AC) and six cycles of CMF in 2008 node-negative, ER-negative patients (Fisher *et al*, 2001).

Summing up the current evidence, it appears that only two class of anthracycline-based regimens have provided sufficient indication of their superiority over CMF: those with three drugs (FAC or FEC like) and the sequential anthracycline → CMF regimens (like the one that we used in the MAM1 trial), while for two-drug regimens, they rather seem equivalent to CMF. This should be adequately considered both in the clinical practice and in the research setting. In particular, at this latter regard, the interpretation of the results of taxane-based adjuvant regimens should carefully consider the type of anthracycline-based regimen employed as a reference arm.

The optimisation of the adjuvant strategy in premenopausal women with endocrine-responsive disease represents a very intriguing task owing to the availability of various alternative and potentially complementary therapeutic options, including chemotherapy, tamoxifen and ovarian suppression. Indirect comparisons within EBCCTG overview (1996; 2000b) suggest a similar benefit in terms of the reduction of recurrence and death risk, form adjuvant ovarian ablation (by surgical oophorectomy or ovarian irradiation), chemotherapy or, for ER+ patients, from tamoxifen. Direct comparisons within the meta-analysis also indicate that the effect of chemotherapy is independent of (and additive with) the effect of tamoxifen, in women aged ⩽50 or >50 years, thus suggesting that the administration of chemotherapy and tamoxifen should be the standard treatment for the majority of women with endocrine-responsive tumours. More controversial remains the role of ovarian ablation. Recently, the results of five comparative trials of adjuvant hormonal therapy using GnRH agonists alone (Schmid *et al*, 2002; Kaufmann *et al*, 2003) or in combination with tamoxifen (Boccardo *et al*, 2000; Roche *et al*, 2000; Jakesz *et al*, 2001), *vs* cytotoxic chemotherapy have shown at least equivalence of effect in premenopausal women with ER-positive tumours; therefore, the ovarian suppression may represent a valid alternative to chemotherapy at least for patients with low–moderate risk. The main question now is whether there is an additional benefit from adding the suppression of ovarian function, either alone or in combination with tamoxifen, to patients who also receive chemotherapy. In the MAM1 trial presented here, we show how the addition of a combination of goserelin plus tamoxifen for 2 years after chemotherapy, for ER-unselected premenopausal patients, yields a further statistically significant reduction in the hazard of relapse (HR=0.74). Also, a trend to a reduction in the hazard of death was observed (HR=0.84), although nonstatistically significant. The effect appears unrelated to the type of chemotherapy (A → CMF or CMF) administered, as no interaction was observed between the two factors of the study design. With regard to the endocrine treatment, however, some limitations of our trial deserve discussion. First, about 21% of patients were ER negative and as much as 42% had unknown ER status. Second, about 30% of patients randomised to goserelin plus tamoxifen did not complete the endocrine therapy as per protocol (Figure 1). Both these issues may have reduced the magnitude of the benefit from the endocrine treatment. Third, a small, and nonstatistically significant, imbalance in the distribution of ER-negative patients, which appear more frequent in the chemotherapy-only arms (Table 1), may have favoured the endocrine treatment. However, this should be a minor issue since the benefits of the endocrine treatment persist at multivariate analysis, where adjustment by receptor status along with other major prognosticators was carried out. Fourth, in our study, tamoxifen was administered for 2 years only, while the optimal duration of such a treatment is now known to be 5 years. Finally, the incidence of chemotherapy-induced amenorrhoea was not recorded in our study and this did not allow testing for an interaction between the persistence of menses and the effect of goserelin plus tamoxifen.

Results similar to ours have already been reported by others in a preliminary manner. The Zoladex In Premenopausal Patients (ZIPP) trial determined the effect of adding goserelin to standard adjuvant treatment (surgery±radiotherapy±chemotherapy±tamoxifen) in women <50 years of age. At a median follow-up of 66 months, the addition of goserelin reduced both the hazard of recurrence (HR=0.80; 95% CI: 0.7–0.92; *P*<0.001) and death (HR=0.82; 95% CI: 0.67–0.99; *P*=0.04). Subgroup analysis suggested that goserelin had its greatest effect in patients with ER-positive tumours who did not receive chemotherapy, but none of the tests for interaction were significant (Baum *et al*, 2001). A study (INT-0101) by the Easter Cooperative Oncology Group (ECOG)/South West Oncology Group (SWOG)/CALGB (Cancer and Leukaemia Group B) (Davidson *et al*, 1999) in node-positive, ER-positive patients compared the addition of goserelin with or without tamoxifen to CAF. At a median follow-up of 6.2 years, there was a trend in favour of improved DFS for the addition of goserelin to CAF, although this just failed to reach statistical significance. The addition of goserelin plus tamoxifen to CAF chemotherapy resulted in a significant benefit in DFS over the use of CAF plus goserelin (*P*<0.01). In this study, the addition of tamoxifen seemed to be more efficacious in those women with postmenopausal oestradiol (E2)levels at the end of adjuvant CAF therapy, while goserelin was more beneficial in women with premenopausal oestradiol levels at the end of CAF therapy.

In summary, the results of our trial and of INT-0101 trial indicate an additional benefit from adding goserelin plus tamoxifen after chemotherapy, for moderate-to-high-risk (node+) patients. Similarly, according to the ZIPP trial, the addition of goserelin alone may yield an additional benefit as compared to chemotherapy alone. However, there are no data evaluating the worth of inducing ovarian suppression to patients who also receive tamoxifen after chemotherapy and this question remains a matter of research.

## Figures and Tables

**Table 1:** Main pretreatment characteristics

	**CMF**		**CMF → GT**		**A → CMF**		**A → CMF → GT**	
**No. of patients**	**114**		**120**		**119**		**113**	
*Tumour size*								
Median (cm)	2		2.2		2.5		2.5	
Range (cm)	0.5–10		1–8		0.18–20		0.7–9.5	
				
*Age (years)*								
Median	45		44		43		44	
Range	29–52		28–51		20–51		25–50	
				
*ER receptor*								
Positive	49	43%	41	34%	37	31%	48	42%
Negative	30	26%	27	23%	24	20%	15	13%
Unknown	35	31%	52	43%	58	49%	50	44%
								
*PgR receptor*								
Positive	41	36%	37	31%	35	29%	41	36%
Negative	33	29%	24	20%	20	17%	19	17%
Unknown	40	35%	59	49%	64	54%	53	47%
								
*Node metastases*								
1–3	64	56%	64	53%	64	54%	61	54%
4+	50	44%	56	46%	55	46%	52	46%
								
*Grading*								
1	3	3%	3	3%	4	3%	1	1%
2	27	24%	24	20%	30	25%	23	20%
3	44	39%	50	42%	43	36%	50	44%
Unknown	40	35%	43	36%	42	35%	39	35%

CMF=CMF × 6 cycles; CMF → GT=CMF × 6 cycles followed by goserelin plus tamoxifen × 2 years; A → CMF=doxorubicin × 4 cycles followed by CMF × 6 cycles; A → CMF → GT=doxorubicin × 4 cycles followed by CMF × 6 cycles followed by goserelin plus tamoxifen × 2 years; ER receptor=oestrogen receptor; PgR receptor=progesterone receptor; CMF=cyclophosphamide, methotrexate, fluorouracil.

**Table 2:** Incidence of grade 3–4 toxic events by study arm on a per patient basis

	**CMF**		**CMF → GT**		**A → CMF**		**A → CMF → GT**	
**Patients**	**114**		**120**		**119**		**113**	
Alopecia	1	0.9%	3	2.5%	28	23.5%	42	37.2%
Astenia	0	0.0%	0	0.0%	0	0.0%	0	0.0%
Cystitis	0	0.0%	0	0.0%	1	0.8%	0	0.0%
Dermatologic	1	0.9%	1	0.8%	0	0.0%	0	0.0%
Diarrhoea	1	0.9%	0	0.0%	1	0.8%	1	0.9%
Fever	0	0.0%	0	0.0%	0	0.0%	0	0.0%
Gastrointestinal	2	1.8%	0	0.0%	1	0.8%	1	0.9%
Liver	0	0.0%	0	0.0%	0	0.0%	0	0.0%
Mucositis	0	0.0%	1	0.8%	0	0.0%	0	0.0%
Vomiting	4	3.5%	5	4.2%	10	8.4%	9	8.0%
Others	0	0.0%	1	0.8%	0	0.0%	0	0.0%
								
Anaemia	1	0.9%	0	0.0%	1	0.8%	0	0.0%
Leuco/neutropenia	5	4.4%	4	3.3%	5	4.2%	5	4.4%
Piastrinopenia	0	0.0%	0	0.0%	1	0.8%	0	0.0%

CMF=CMF × 6 cycles; CMF → GT=CMF × 6 cycles followed by goserelin plus tamoxifen × 2 years; A → CMF=doxorubicin × 4 cycles followed by CMF × 6 cycles; A → CMF → GT= doxorubicin × 4 cycles followed by CMF × 6 cycles followed by goserelin plus tamoxifen × 2 years; CMF=cyclophosphamide, methotrexate, fluorouracil.

## Appendix A

**Complete list of coauthors** **Università Federico II, Napoli 80131** Sabino De Placido, Michele De Laurentiis, Angelo Raffaele Bianco, Rossella Lauria, Clorindo Pagliarulo, Alfredo Marinelli, Chiara Carlomagno, Giuseppe Petrella, Gennaro Limite, Francesca Caputo

**Istituto Oncologico, Bari 70100** Mario De Lena, Vito Lorusso, Angelo Paradiso, Michele Guida, Agnese Latorre, Antonio Mazzei, Francesco Schittulli, Cosimo D'Amico

**Università di Sassari, Sassari 07100** Antonio Farris, Maria Giuseppina Sarobba, Giovanni Sanna, Maria Grazia Alicicco, Giovanna Fois

**Ospedale S. Maria Goretti, Latina 04100** Modesto D'Aprile, Giorgio Pistillucci, Maurilio Natali, Susanna Busco, Francesco Angelini

**Istituto Oncologico Santi Currò, Catania 95100** Francesco Ferraù, Giuseppe Failla

**Azienda Ospedaliera Mariano Santo, Cosenza 87100** Salvatore Palazzo, Virginia Liguori, Antonio Roviti

**Università di Palermo, Palermo 90100** Sergio Palmeri, Marina Vaglica, Cristina Raimondi

**Azienda Ospedaliera S. Carlo, Potenza 85100** Luigi Manzione, Domenico Bilancia, Rosangela Romano

**Policlinico Universitario, Messina 98100** Vincenzo Adamo, Giuseppe Altavilla, Claudia Garipoli

**Ospedale Civile, Lamezia Terme 88046** Ettore Greco

**Azienda Ospedaliera Umberto I, Enna 94100** Rosalia Carroccio

**Istituto Regina Elena, Roma 00144** Francesco Cognetti

**Università – Policlinico Le Scotte, Siena 53100** Guido Francini

**Ospedale Fatebenefratelli, Benevento 82100** Tonino Pedicini, Antonio Febbraro, Claudia Corbo

**Università Gabriele D'Annunzio, Chieti 66100** Stefano Iacobelli

**Data Management** Raffaele Costanzo, Diego D'Agostino, Valeria Forestieri, Ciro Gallo, Francesco Perrone

**Pharmacia Corporation** Francesco Marra
